# Factors associated with early discontinuation of anamorelin in patients with cancer-associated cachexia

**DOI:** 10.1007/s00520-023-08097-4

**Published:** 2023-10-10

**Authors:** Ikuto Tsukiyama, Takashi Iwata, Tomoko Takeuchi, Ryoko Inuzuka Kato, Masaki Sakuma, Sumiyo Tsukiyama, Mana Kato, Yoshiaki Ikeda, Wataru Ohashi, Akihito Kubo, Naoharu Mori

**Affiliations:** 1 Clinical Onco-pharmacology, Faculty of Pharmacy, Meijo University, 150 Yagotoyama, Tempaku-ku, Nagoya, Aichi 468-8503 Japan; 2 Department of Palliative and Supportive Medicine, Graduate School of Medicine, Aichi Medical University, Nagakute, Japan; 3 Department of Pharmacy, Aichi Medical University Hospital, Nagakute, Aichi Japan; 4 Clinical OMICs and Translational Research Center, Meijo University, Nagoya, Aichi Japan; 5 Oncology Center, Aichi Medical University School of Medicine and Hospital, Nagakute, Aichi Japan; 6 Department of Nutrition, Aichi Medical University Hospital, Nagakute, Aichi Japan; 7 College of Pharmacy, Kinjo Gakuin University, Nagoya, Aichi Japan; 8 Division of Biostatistics, Clinical Research Center, Aichi Medical University School of Medicine and Hospital, Nagakute, Aichi Japan

**Keywords:** Cancer-associated cachexia, Anamorelin, Early discontinuation, Risk factor, Performance status, Prognostic nutrition index

## Abstract

**Purpose:**

Cancer-associated cachexia, a multifactorial syndrome involving loss of muscle mass and anorexia, is an unremitting problem for cancer patients. Anamorelin has become available for cancer-associated cachexia, but early discontinuation is common in clinical practice. This study aimed to explore factors related to the early discontinuation of anamorelin and its relationship to survival.

**Patients and methods:**

This prospective, observational study of multimodal clinical practice involved patients who took anamorelin (100 mg) for cancer-associated cachexia at Aichi Medical University Hospital between 14 May 2021 and 31 March 2022. In July 2022, clinical data were extracted from electronic clinical records. Patients who discontinued anamorelin less than 4 weeks after initiation were defined as the early discontinuation group, and their clinical data and survival time were compared with those of the continuation group. This study was approved by the Ethics Committee of the university (approval no. 2021-124).

**Results:**

Of the 42 patients treated with anamorelin, 40 (median age 72.5 years, median BMI 18.7 kg/m^2^) were analyzed, including 13 with non-small cell lung cancer, and 12 with pancreatic, 8 with colorectal, and 7 with gastric cancers. On univariate analysis, the early discontinuation group included more patients with worse performance status (PS) (*p*=0.028), low prognostic nutritional index (PNI) (*p*=0.001), and no concomitant anticancer drugs (*p*=0.003). On multivariate analysis, PS and PNI were related to anamorelin continuation. Survival time was significantly shorter in the early discontinuation group (*p*=0.039).

**Conclusion:**

Worse PS and low PNI were associated with early discontinuation of anamorelin. Longer survival time was observed in the continuation group.

## Introduction

Cancer-associated cachexia is a multifactorial wasting syndrome characterized by inflammation-associated loss of muscle mass and anorexia that affects approximately 70% of patients with pancreatic cancer, 60% of patients with gastroesophageal cancer, and 50% of patients with lung and colorectal cancers [1]. Warren reported that cancer-associated cachexia affects the survival of cancer patients and is implicated in 20% of all cancer deaths in 1932 [2]. Cancer-associated cachexia was defined by Fearon et al. in 2010 as “a multifactorial syndrome characterized by a persistent loss of skeletal muscle mass (with or without fat loss) that cannot be completely reversed by conventional nutritional therapy and results in progressive functional impairment” [3]. Various approaches have been tried, including nutritional therapy and drug treatment, but no effective treatment has yet been established [4–8]. In January 2021, anamorelin, a novel medicine for cancer-associated cachexia, was approved by the Ministry of Health, Labour and Welfare in Japan, although it had not yet been approved by the Food and Drug Administration in the USA and the European Medicines Agency [9, 10]. Anamorelin is a ghrelin-like agonist that binds to the growth hormone (GH)–releasing factor receptor type 1a (GHS-R1a), and it is thought to act by a mechanism similar to that of ghrelin. Its binding to GHS-R1a stimulates the release of GH in the pituitary gland and increased appetite in the hypothalamus [11]. GH release from the pituitary gland promotes muscle protein synthesis via IGF-1 secretion from the liver [12]. It was shown that anamorelin not only improved appetite, but also increased lean body mass in a phase 3 clinical trial in patients with non-small cell lung cancer (NSCLC) and a phase 2 trial in patients with gastric, pancreatic, and colorectal cancers [13, 14]. In these two clinical trials, 66% and 62% of the patients completed the planned 12-week anamorelin treatment periods, respectively. ROMANA3, an international phase 3 clinical trial in patients with non-small cell lung cancer, reported that 64% of the patients in the anamorelin group completed the scheduled 24-week anamorelin treatment, and it was well tolerated [15]. An interim report from the post-marketing surveillance by the manufacturer of anamorelin notably indicates that more than 30% of patients treated with anamorelin discontinued it early, within 3 weeks. In real-world clinical practice, not a small number of patients discontinue it early, and factors affecting its continuation are unknown. The purpose of this study was to explore factors related to early anamorelin discontinuation.

## Methods

### Patients

Patients aged 20 years or older, able to use the Japanese language, and receiving anamorelin for cancer-associated cachexia in the Palliative Care Center or Oncology Center, or the Departments of Gastrointestinal Medicine or Respiratory Medicine and Allergology in Aichi Medical University School of Medicine and Hospital, and who had one of colorectal cancer, lung cancer, gastric cancer, or pancreatic cancer were eligible. Patients who were unable to consent to the treatment plan of their own volition due to cognitive decline were excluded.

### Study design

This single-center, prospective, observational study was conducted after approval was obtained from the Institutional Review Board of Aichi Medical University School of Medicine and Hospital. A primary objective of this study was to explore potential factors related to the early discontinuation of anamorelin. A secondary objective was to assess improvement of symptoms related to cachexia by anamorelin treatment, and the influence of the early discontinuation on survival.

Patients who discontinued anamorelin within 4 weeks from its start were classified as the early discontinuation group, whereas those who continued it beyond 4 weeks were classified as the continuation group. Background demographic characteristics and laboratory values at the onset of anamorelin treatment were compared between the early discontinuation and continuation groups. The anamorelin package insert states, “If weight gain or improvement in appetite is not observed, it should be withdrawn in principle after approximately 3 weeks from the onset of its administration” [16]. A 1-week lag in the evaluation time point was allowed to accommodate for real-world clinical practice in the present study, given that patients visit the hospital to receive chemotherapy not only every 3 weeks, but also every 2 or 4 weeks.

### Intervention and service model

In the outpatient clinic of the Palliative Care Center in this hospital, the patient can be seen and receive multidisciplinary care from a medical team composed of several medical professionals including a physician, a nurse, a pharmacist, a nutritionist, and a dental hygienist. Patients who received anamorelin met a nutritionist and received dietary advice. Appropriate rehabilitation or physical training was suggested by the attending physician or palliative care physician based on each patient’s food intake. Dietary advice and physical training were continued at a degree appropriate for each patient, regardless of the discontinuation of anamorelin. Patients took 100 mg of anamorelin once a day while fasting, and symptoms and adverse events were recorded in an anamorelin diary from the onset of the treatment. Any treatment for primary cancer or a metastatic site, pain, other cancer-related symptoms, and other underlying diseases was permitted.

### Data collection

Background demographic characteristics, physical examination findings, subjective symptoms, laboratory values, and concomitant medications of patients before administration of anamorelin were extracted from the clinical medical record and diary. The Glasgow prognostic score (GPS) was calculated according to McMillan et al.: 0, serum albumin (Alb) ≥ 3.5 g/dL and C-reactive protein (CRP) < 1 mg/dL; 1, Alb < 3.5 g/dL or CRP ≥ 1 mg/dL; and 2, Alb < 3.5 g/dL and CRP ≥ 1 mg/dL [17]. The prognostic nutritional index (PNI) was calculated according to the following formula as reported by Onodera et al: PNI = 10 × Alb (g/dL) + 0.005 × total lymphocyte count (TLC) (/mm^3^) [18]. Patients were followed for 1 year from the onset of anamorelin treatment or until the date of death for this analysis.

### Statistical analysis

All patients who received anamorelin during the study period were included in this study. Fisher’s exact test was used to compare the prevalence of patients with demographic factors at baseline between the early discontinuation and continuation groups. The Mann-Whitney *U* test was used to compare laboratory values between groups. Multivariate logistic regression analysis was used to evaluate the associations between early discontinuation and background factors. The multivariate analyses were performed using the forced entry method with the items that showed *p* < 0.05 on the univariate analysis entered as explanatory variables in principle, and 95% confidence intervals were calculated. Confounding factors and items with median values that were slightly significant and within the normal range were excluded from the explanatory variables. Eventually, given the small number of cases in this study, Eastern Cooperative Oncology Group performance status (ECOG-PS) and PNI were entered as explanatory variables, with anamorelin early discontinuation as the dependent variable, and with anticancer drugs as a covariate in the analysis. Serum albumin and GPS were excluded from the explanatory variables due to confounding factors with PNI, and white blood cell count, absolute neutrophil count, and serum sodium were also excluded due to the median being within the normal range in both groups. Receiver operating characteristic (ROC) analysis was performed to identify the cut-off values of PS and PNI. Duration of treatment and survival were analyzed by the Kaplan–Meier method with the log-rank test. For all analyses, *p* < 0.05 was set as the significance level. IBM SPSS Statistics for Windows, version 28 (IBM Corp, Armonk, NY) was used for all statistical procedures.

### Ethical considerations

This study was approved by the ethics review board of Aichi Medical University (approval no. 2021-124), and opt-out consent was offered with the permission of the board because no intervention beyond routine clinical practice was included. This study was conducted according to the Declaration of Helsinki and the ethical guidelines regarding life science and medical studies of humans in Japan.

## Results

Forty-two patients received anamorelin from May 1, 2021, to March 31, 2022. Forty cases were included in the analysis, and two were not included due to missing data. The background characteristics of the patients are shown in Table 1. In brief, there were 15 females and 25 males with a median age of 72.5 (range 32–83) years and a median body mass index (BMI) of 18.7 (range 12.9–23.8) kg/m^2^. The patients’ diagnoses were NSCLC (*n* = 13), pancreatic cancer (*n* = 12), colorectal cancer (*n* = 8), and gastric cancer (*n* = 7), and they had a PS at baseline of 0 (*n* = 9), 1 (*n* = 15), 2 (*n* = 7), 3 (*n* = 7), and 4 (*n* = 2).

**Table 1:** Patients’ background characteristics and results of univariate analysis

	Total (*n* = 40)	Early discontinuation (≤ 4 weeks) (*n* = 19)	Continuation (> 4 weeks) (*n* = 21)	*p*-value
	Number of patients (%)	Number of patients (%)	Number of patients (%)	
Sex				
Male	25 (62.5)	11 (57.9)	14 (66.7)	0.745
Female	15 (37.5)	8 (42.1)	7 (33.3)	
Performance status (ECOG)				
0	9 (22.5)	1 (5.3)	8 (38.1)	0.028
1	15 (37.5)	6 (31.6)	9 (42.9)	
2	7 (17.5)	5 (26.3)	2 (9.5)	
3	7 (17.5)	5 (26.3)	2 (9.5)	
4	2 (5)	2 (10.5)	0 (0)	
Primary site of cancer				
Lung (NSCLC)	13 (32.5)	9 (47.4)	4 (19.0)	0.189
Pancreas	12 (30.0)	6 (31.6)	6 (28.6)	
Colorectum	8 (20.0)	2 (10.5)	6 (28.6)	
Gastric	7 (17.5)	2 (10.5)	5 (23.8)	
Stage				
III	4 (10.0)	2 (10.5)	2 (9.5)	1.000
IV	36 (90.0)	17 (89.5)	19 (90.5)	
Site of metastasis				
Liver	10 (25.0)	6 (31.6)	4 (21.1)	0.473
Bone	8 (20.0)	5 (26.3)	3 (15.8)	0.442
Peritoneum	8 (20.0)	4 (21.1)	4 (21.1)	1.000
Lung	7 (17.5)	2 (10.5)	5 (26.3)	0.412
Brain	5 (12.5)	2 (10.5)	3 (15.8)	1.000
Drug				
Anticancer drug	29 (72.5)	9 (47.4)	20 (95.2)	0.001
Opioid	11 (27.5)	9 (47.4)	2 (9.5)	0.012
GPS				
0	13 (32.5)	3 (15.8)	10 (47.6)	0.018
1	9 (22.5)	3 (15.8)	6 (28.6)	
2	18 (45.0)	13 (68.4)	5 (23.8)	
Symptom				
Appetite loss	38 (95.0)	19 (100)	19 (90.5)	0.488
Fatigue	28 (70.0)	14 (73.7)	14 (66.7)	0.736
Nutritionist consultation	35 (87.5)	17 (89.5)	18 (85.7)	1.000
	Median (range)	Median (range)	Median (range)	
Age (y)	72.5 (32–83)	74 (51–83)	71 (32–80)	0.091
Body weight (kg)	49.4 (27.8–66.7)	48.7 (27.8–60.1)	50.8 (37.0–63.7)	0.448
Body mass index (kg/m^2^)	18.7 (12.9–23.8)	18.8 (12.9–22.7)	18.6 (14.9–23.8)	0.915
Laboratory data				
AST (U/L)	22 (6–77)	19 (6–53)	30 (10–77)	0.150
ALT (U/L)	16 (4–47)	12 (4–43)	19 (7–47)	0.066
ALP (U/L)	103 (13–543)	102 (48–543)	108 (13–443)	0.851
LDH (U/L)	191 (111–443)	201 (120–387)	190 (111–443)	0.524
Scr (mg/dL)	0.69 (0.35–4.74)	0.70 (0.35–4.74)	0.68 (0.41–1.35)	0.509
Ccr (mL/min)	66.6 (9–163)	64.1 (9–163)	70.3 (26–119)	0.196
WBC (/μL)	6800 (2400–17,000)	8300 (3500–17,000)	5900 (2400–14,800)	0.005
ANC (/μL)	4762 (1044–23,424)	6329 (1309–23,424)	3416 (1044–13,246)	0.004
TLC (/μL)	1156 (170–2200)	1030 (170–2170)	1180 (810–2200)	0.486
Na (mEq/L)	139 (127–144)	136 (127–142)	140 (131–144)	0.007
Ca (adjusted) (mg/dL)	8.7 (8.5–11.2)	9.6 (8.5–11.2)	9.4 (9.1–10.1)	0.958
Glucose (mg/dL)	114 (56–302)	123 (56–302)	112.5 (84–228)	0.744
Alb (g/dL)	3.3 (1.3–4.5)	2.8 (1.3–3.9)	3.5 (2.2–4.5)	0.001
CRP (mg/dL)	1.09 (0.03–14.0)	3.84 (0.05–13.3)	0.49 (0.03–14.0)	0.008
PNI	33 (13–45)	28 (13–39)	35 (22–45)	0.001

*GPS* Glasgow prognostic score, serum albumin > 3.5 and CRP < 1: 0, serum albumin ≤ 3.5 and CRP > 1, other: 1

*PNI*: prognostic nutritional index (%), Alb (g/L) + 0.005 × TLC (count/μL)

Early discontinuation (≤ 4 weeks): discontinued anamorelin within 4 weeks, continuation (> 4 weeks): continued anamorelin over 4 weeks

*ECOG* Eastern Cooperative Oncology Group, *NSCLC* non-small cell lung cancer, *AST* aspartate aminotransferase, *ALT* alanine aminotransferase, *ALP* alkaline phosphatase, *LDH* lactate dehydrogenase, *Scr* serum creatinine, *Ccr* creatinine clearance, *WBC* white blood cell count, *ANC* absolute neutrophil count, *TLC* total lymphocyte count, *Na* serum sodium, *Ca* serum calcium, *Alb* serum albumin, *CRP* C-reactive protein

**Fig. 1:**
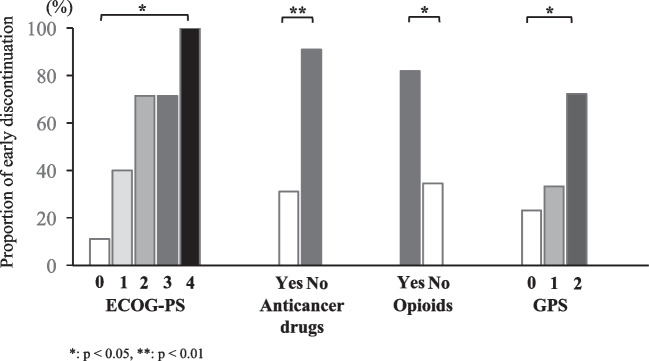
Proportion of patients with early discontinuation of anamorelin. *p < 0.05, **p < 0.01 by Fisher’s exact test

The proportion with early discontinuation of anamorelin increased with higher PS and GPS (Fig. 1). The rate of early discontinuation was significantly higher for patients with PS 2–4 than for patients with PS 0–1 (75% vs 29%, *p* < 0.001). In the patients who received concomitant anticancer drugs, the rate of early discontinuation was significantly lower than in those without concomitant anticancer drugs (31% vs 90%, *p* = 0.001). The early discontinuation rate was significantly higher in patients taking opioid analgesics than in those not taking them (82% vs 35%, *p* = 0.012). In addition, the early discontinuation rate was significantly higher in patients with GPS 2 than in those with GPS 0–1 (72% vs 27%, *p* = 0.01).

On univariate analysis, the early discontinuation group included significantly more patients with PS 2–4 and taking concomitant opioids and fewer taking concomitant anticancer drugs than the continuation group (Table 1). In the early discontinuation group, CRP, leukocytes, and neutrophil counts were significantly higher, and serum sodium, serum albumin, and PNI were significantly lower than in the continuation group. Multivariate analysis was conducted by logistic regression analysis with the cut-off levels of PS 1 and PNI 30, which were identified by ROC analysis. PS and PNI were identified as factors related to anamorelin continuation; PS 2–4 and PNI < 30 were associated with early discontinuation of anamorelin (Table 2).

**Table 2:** Multivariate analysis for predictors of anamorelin discontinuation

	Odds ratio	95% CI	*p*-value
ECOG-PS ≥ 2	7.80	(1.065–57.13)	0.043
PNI ≥ 30	0.05	(0.005–0.386)	0.005
Anticancer drugs	0.12	(0.008–1.697)	0.116

Adverse events were the primary reason for the discontinuation of anamorelin (*n* = 15), followed by dysphagia (*n* = 7), and insufficient efficacy was the next major reason for discontinuation, observed only in the early discontinuation group (*n* = 5; Table 3). The most common adverse event causing discontinuation of anamorelin was hyperglycemia, followed by gastrointestinal symptoms such as nausea and diarrhea. In terms of efficacy, appetite improved in 90% of the patients in the continuation group, but in only 26% of the patients in the early discontinuation group (*p* < 0.001; Table 3).

**Table 3:** Symptom improvement with anamorelin treatment and reasons for discontinuation

	Early discontinuation (≤4 weeks) (*n* = 19)	Continuation (>4 weeks) (*n* = 21)	*p*-value
	Number of patients (%)	Number of patients (%)	
Symptom improvement with anamorelin treatment			
Appetite improvement	5 (26.3)	19 (90.5)	<0.001
Body weight gained	9 (47.4)	10 (47.6)	1
Fatigue/malaise improved	3 (15.8)	5 (23.8)	0.698
Either	14 (73.7)	20 (95.2)	0.085
Reasons for discontinuation of anamorelin			
Adverse events	9 (47.4)	6 (28.6)	0.328
Hyperglycemia	2 (10.5)	3 (14.3)	1
Nausea	1 (5.3)	2 (9.5)	1
Diarrhea	2 (10.5)	0 (0)	0.219
Cardiac conduction disturbances	2 (10.5)	0 (0)	0.219
Hepatic disorder	0 (0)	1 (4.8)	1
Headache	1 (5.3)	0 (0)	0.475
Numbness of the tongue	1 (5.3)	0 (0)	0.475
Perspiration	1 (5.3)	0 (0)	0.475
Cough	1 (5.3)	0 (0)	0.475
Dysphagia	2 (10.5)	5 (23.8)	0.412
Insufficient efficacy	5 (26.3)	0 (0)	0.018
Improved appetite	0 (0)	3 (14.3)	0.233
Treated for 12 weeks	0 (0)	2 (9.5)	0.488
Changed hospital	1 (5.3)	0 (0)	0.475
Died	0 (0)	1 (4.8)	1
Others	2 (10.5)	1 (4.8)	0.596

**Fig. 2:**
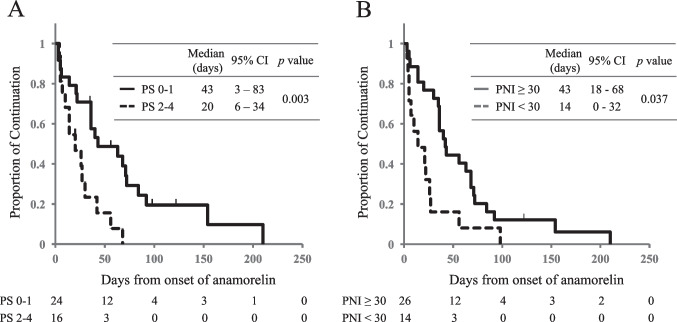
Time to discontinuation of anamorelin. Kaplan–Meier estimates of time to discontinuation of anamorelin treatment based on the classification according to **A** PS and **B** PNI. PS, Eastern Cooperative Oncology Group performance status; PNI, prognostic nutritional index; CI, confidence interval

A shorter duration of anamorelin treatment was seen in patients with PS 2–4 compared with those with PS 0–1 (20 days vs 43 days, *p* = 0.003; Fig. 2A). Patients with PNI < 30 discontinued anamorelin significantly earlier than those with PNI ≥ 30 (14 days vs 43 days, *p* = 0.037; Fig. 2B).

**Fig. 3:**
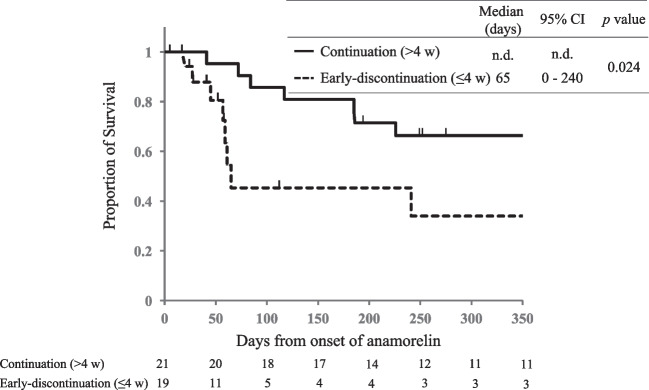
Anamorelin continuation and survival. Kaplan–Meier estimates of overall survival from the onset of anamorelin treatment in the continuation and early discontinuation group. CI, confidence interval; n.d., not detected

The duration of survival from the start of anamorelin was significantly longer in the continuation group than in the early discontinuation group (not reached vs 65 days, *p* = 0.01; Fig. 3).

## Discussion

This study explored potential predictors of early discontinuation of anamorelin among baseline demographic characteristics and laboratory values in patients who took anamorelin in clinical practice. It was found that poor PS and low PNI were risk factors for early discontinuation of anamorelin, and shorter survival was observed in patients who discontinued anamorelin early. To the best of our knowledge, this is the first report to identify factors related to the continuation of anamorelin treatment in the context of multimodal clinical practice including nutritional support, and it is expected that these results will contribute to the appropriate use of anamorelin.

First, for the primary objective, good PS and high PNI were found to have a positive association with anamorelin continuation. The domestic phase 2, phase 3, and international phase 3 clinical trials of anamorelin, all of which included patients with PS ≤ 2 and a prognosis of at least 4 months, reported that 62–66% of participants completed the planned 12 weeks or 24 weeks of anamorelin treatment, and that it was well tolerated [13, 14]. In the present study, a quarter of the patients were in poor general condition with PS 3 or 4, and 7 of these 9 patients discontinued anamorelin early. PS is known to be a prognostic factor in cancer treatment, and patients with poor PS are known to have shorter survival [19–21]. This may have affected the shorter duration of anamorelin treatment in patients with poor PS. The reasons for half of the early discontinuation cases were adverse events, and a quarter of the cases cited discontinuation due to lack of efficacy in the early discontinuation group. In the domestic phase 3 clinical trial in NSCLC patients, only 3.6% of patients in the anamorelin group discontinued the drug due to adverse events [13]. Although it cannot be simply compared, the proportion of discontinuation due to adverse events in the present study was higher, at 47%, than that reported in the clinical trial. In a meta-analysis that integrated four clinical trials of chemotherapy for patients with metastatic colorectal cancer, poor PS was reported as a risk factor for severe toxicity of anticancer drugs [22]. In this study, nine patients (23%) with PS 3–4 were included, and all of them developed one or more adverse events, which might have been related to the increased incidence of adverse events. Withdrawal of anamorelin due to lack of efficacy occurred in 26% of patients in the early discontinuation group, but this was not observed in the continuation group. Of the nine patients with PS 3–4, improvement of appetite, malaise, and/or fatigue was observed in three cases, suggesting that anamorelin is less effective and causes more adverse events in patients with poor PS. In addition, worse PS at baseline was correlated with an increase in the proportion of early discontinuation (Fig. 1) and shorter duration of anamorelin treatment (Fig. 2A). On the other hand, one patient with PS 3 had improved appetite and was able to continue anamorelin without adverse events, and this patient had a PNI > 30. Five patients (26%) had improved appetite and three patients (16%) had improved malaise and/or fatigue in the early discontinuation group. It was suggested that there may be patients with poor PS who could benefit from anamorelin, and it is necessary to identify indicators for such patients.

The PNI is calculated by adding serum albumin and the total lymphocyte count, and it is thought to reflect inflammation and immune status. It is reported that a low PNI contributes to shorter survival in patients with pancreatic cancer, lung cancer, and gastric cancer [23–25]. Low pretreatment PNI has also been reported to correlate with worse survival in patients with advanced NSCLC and gastric cancer who received chemotherapy [26, 27]. In the present study, a low PNI prior to anamorelin initiation was identified as a risk factor for early discontinuation of the treatment. There was no correlation between the PNI and the occurrence of adverse events, but patients with low PNI levels had a lower rate of appetite improvement than those with high PNI levels (36% vs 73%, *p* = 0.041). To improve appetite with anamorelin, it may be more appropriate to start anamorelin in the early stages of cancer-associated cachexia, when the decreases in albumin and the lymphocyte count are less severe.

Although more patients had received concomitant anticancer drugs in the continuation group than in the early discontinuation group, this factor was input as a covariate in the multivariate analysis. The main reason for this was that the patients suitable for anticancer drugs were those with good PS, and thus, the concomitant use of anticancer drugs was considered a potential covariate with PS. Some adverse events of anticancer drugs overlap with symptoms of cancer-associated cachexia, such as nausea and vomiting, fatigue, and malaise, which may have potentially counteracted the effect of anamorelin. However, appetite improved in 19 (66%) of the 29 patients who were receiving anticancer drugs at the time of anamorelin initiation, and fatigue and malaise improved in five patients (17%). In contrast, appetite improved in only 3 (27%) of 11 patients who had not received anticancer drugs at the onset of anamorelin, and fatigue and malaise improved in 1 patient (9%). In the domestic phase 2 and 3 clinical trials of anamorelin, more than 80% of eligible patients had been treated with concomitant anticancer drugs and had shown improved appetite and increased lean body mass [13, 14]. If tumor growth is controlled by anticancer drugs, and survival is prolonged, the duration of benefit from anamorelin may be extended accordingly. It is also possible that anticancer drugs may have been improving the patient’s general condition independently of anamorelin due to the treatment of the cancer itself. Mitsunaga et al. reported that the incidence of cachexia in advanced pancreatic cancer was 50% at the start of first-line chemotherapy [28]. They also reported that the remaining 32% of patients developed cachexia within 3 months of the chemotherapy and suggested that, eventually, all studied patients might experience cachexia. Patients undergoing anticancer treatment who have a good PS should be suitable candidates for anamorelin treatment, and early screening for cachexia and introduction of anamorelin should be considered. Early discontinuation was more common in patients taking concomitant opioids than in those not taking them, but there was no difference in the duration of anamorelin treatment with or without concomitant opioid use (data not shown). The two patients who continued anamorelin with opioids both had PS0 and PNI > 30, and their appetite improved with anamorelin. In contrast, 9 of 10 patients who did not receive any opioids and discontinued anamorelin early had PS ≥ 2 or PNI < 30. Of these, five discontinued due to adverse events and two due to insufficient response. It was suggested that patients who use opioids could also benefit from anamorelin and that these patients were those with good PS and PNI.

As for the secondary objective, the survival period was longer in the continuation group than in the early discontinuation group. A higher number of patients with good PS and concomitant anticancer drugs were in the continuation group than in the early discontinuation group. This might have led to longer survival in the continuation group. Muscle loss and sarcopenia associated with hypermetabolism due to cachexia have been reported to increase the risk of serious hematological and dose-limiting toxicities and are associated with shorter survival in patients with various types of advanced cancers [29–32]. Improved appetite with anamorelin was observed in 19 of 21 patients in the continuation group based on the nutritionist’s support in this study. This might lead to an improved internal environment that enhanced tolerance and elicited the potential effects of anticancer drugs. Despite this, it is difficult to determine whether anamorelin contributes, albeit indirectly, to prolonging survival of patients with cancer-associated cachexia based solely on the present results. To assess the effect of anamorelin on survival, comparative studies with placebo are needed, and fundamentally, changes in physical function, dietary intake, and quality of life due to anamorelin treatment need to be assessed.

### Limitations

This study has several limitations. First, use of other modalities with anamorelin treatment was not restricted in this study. Cancer-associated cachexia is a multifactorial, metabolic disorder requiring a multimodal approach to treatment that includes not only drugs but also nutritional support and physical exercise. This study was conducted in a setting in which nutritionists provided nutritional support for all patients receiving anamorelin. Although rehabilitation was not performed for all patients, recommendations regarding exercise therapy appropriate to the nutritional status of each patient were given by the palliative nutrition specialist and the attending physician. Therefore, no forceful exercise therapy was given to patients unable to obtain the necessary nutritional intake. Since no problems were observed with the nutritional counseling and physical exercise in combination with anamorelin treatment, these combinations were considered feasible, which may have provided more benefit than anamorelin alone. The nutritional counseling and physical exercise were continued after discontinuation of anamorelin, which might be a source of bias with regard to weight or symptom variation in the patients. Fluid retention might also have affected weight variation.

Second, although discontinuation within 4 weeks was defined as early discontinuation in the present study, many of the patients whose appetite improved experienced the effect earlier, such as a few days to a week after the start of anamorelin. A 1-week later evaluation time point than that indicated in the package insert was allowed to accommodate real-world clinical practice, which may have affected the incidence of early discontinuation or improvement of symptoms as a bias. In addition, this study was based on a small sample size at a single institution, which may have resulted in it being underpowered to detect risk factors. Further investigation based on a larger sample size is warranted to identify the predictors of anamorelin continuation in actual clinical practice.

## Conclusion

Poor PS and low PNI were associated with early discontinuation of anamorelin treatment in clinical practice, and early discontinuation was associated with shorter survival. It is necessary to identify early cancer-associated cachexia before PS and PNI worsen and to apply a multidisciplinary approach that includes anamorelin. However, the present study had a small sample size and was conducted in a single institution, and further investigation is warranted.
